# Anti-Müllerian Hormone Serum Values and Ovarian Reserve: Can It Predict a Decrease in Fertility after Ovarian Stimulation by ART Cycles?

**DOI:** 10.1371/journal.pone.0044571

**Published:** 2012-09-11

**Authors:** Tito Silvio Patrelli, Salvatore Gizzo, Nicoletta Sianesi, Luca Levati, Antonio Pezzuto, Bruno Ferrari, Alberto Bacchi Modena

**Affiliations:** Department of Gynecology, Obstetrics and Neonatology, University of Parma, Parma, Italy; John Hunter Hospital, Australia

## Abstract

**Background:**

A variety of indicators of potentially successful ovarian stimulation cycles are available, including biomarkers such as anti-Mullerian hormone. The aim of our study was to confirm the usefulness of serum anti-Mullerian hormone assay in predicting ovarian response and reproductive outcome in women eligible for ART cycles.

**Materials:**

Forty-six women undergoing ART cycles at the Centre for Reproductive Medicine in Parma were recruited from March-to-June 2010. Inclusion criteria: age<42 years; body-mass-index = 20–25; regular menstrual cycles; basal serum FSH concentration <12 IU/L and basal serum estradiol concentration <70 pg/mL. The couples included in our study reported a variety of primary infertility causes. All women underwent FSH stimulation and pituitary suppression (GnRH-agonist/GnRH-antagonist protocols). Women were considered poor-responders if thay had ≤3 oocytes; normal-responders 4–9 oocytes and high-responders ≥10 oocytes. Serum samples for the AMH assays were obtained on the first and last days of stimulation. A P value ≤0.05 was considered statistically significant.

**Result:**

FSH levels increased significantly when AMH levels decreased. The total dose of r-FSH administered to induce ovulation was not correlated to AMH. The number of follicles on the hCG, serum estradiol levels on the hCG-day, and the number of retrieved oocytes were significantly correlated to AMH. The number of fertilized oocytes was significantly correlated to the AMH levels. No significant correlation was found between obtained embryos or transferred embryos and AMH. Basal serum AMH levels were significantly higher than those measured on the hCG-day, which appeared significantly reduced. There was a significant correlation between AMH in normal responders and AMH in both high and poor responders.

**Conclusions:**

Our data confirm the clinical usefulness of AMH in ART-cycles to customize treatment protocols and suggest the necessity of verifying an eventual permanent decrease in AMH levels after IVF.

## Introduction

Appropriate clinical evaluation and proper treatment of women are essential for a positiveoutcome of assisted reproductive technology (ART) cycles. For good results it is necessary to assess ovarian reserve before planning treatment. The identification of both low and high responders before treatment may reduce cycle cancellation rates and side-effects, such as ovarian hyperstimulation syndrome (OHSS) [1].

Biomarkers with well-understood biological mechanisms and metrics for assay interpretation are needed to provide an ovarian frame for the onset and the end of the menopause transition, as well as to indicate the proximity to the final menstrual period, and to contribute to clinical decision making [2].

For several years, age and day-3 levels of follicle-stimulating hormone (FSH) and luteinizing hormone (LH) have been used as indicators of ovarian response toART.In fact, the basal FSH concentration is the most common test used for ovarian screening [3], however, it has been reported that the increase in FSH levels occurs late in the sequence of events associated with ovarian aging [4]. Therefore, if fertility is considered the end point, this increase may be of limited clinical use as a marker [5]. Recently, several investigators reported the effectiveness of antral follicle count (AFC) and ovarian volume in predicting ovarian response to hormonal stimulation [6], [7]. They stated that AFC provides better prognostic information on the occurrence of poor ovarian response during hormone stimulation for *in vitro* fertilization (IVF) than does the woman’s chronological age or basal FSH. Nonetheless, ultrasoundis subjective, and the interpretation of the observations may not be consistent [8]. So the need persists for a biological endocrine marker that can be used without bias.

Recently, a new endocrine marker, anti-Müllerian hormone (AMH), was evaluated by several study groups as a marker of ovarian response.

AMH is a dimeric glycoprotein member of the transforming growth factor (TGF)-β superfamily. Its most clearly defined role is in male sexual differentiation. AMH is produced by fetal Sertoli cells at the time of testicular differentiation, and induces regression of the Müllerian ducts. In the absence of AMH, the Müllerian ducts develop into the uterus, fallopian tubes and the upper part of the vagina [9]. In women, AMH is produced in the ovary by the granulosa cells surrounding preantral and small antral follicles [10], [11]. AMH expression in ovaries has been observed as early as 36 weeks gestation in humans [12]. Even when using ultrasensitive assays, AMH is barely detectable in the serum at birth. Later, AMH increases after puberty [12], [13] and then declines with advancing female age, to become undetectable again at the time of the menopause [14].

AMH levels correlate well with the number of antral follicles measured by ultrasound [15]–[17] and are believed to be the best representation of the gradual decline in reproductive capacity in women proven to be fertile [18], [19]. Finally, AMH has been shown to be an accurate marker for the occurrence of poor response to ovarian hyperstimulation with gonadotropins in IVF [16], [20], [21]. Based on these findings, AMH may well become a frequently applied marker in reproductive medicine [22].

The purpose of our study was to confirm the usefulness of the serum AMH assay in predicting ovarian response and reproductive outcome in women undergoing ovarian stimulation in ART cycles.

## Methods

For our study we recruited 46 women undergoing ART cycles at the Centre for Reproductive Medicine of the University of Parma - Department of Obstetrics, Gynecology and Neonatology. The study was conducted from 1^st^ March 2010 to 30^th^ June 2010.

All women gave their written informed consent before receiving medical treatment and the study was approved by the University of Parma Ethics Board. Inclusion criteria were: age <42 years; body mass index 20–25; regular menstrual cycles lasting between 26 and 34 days; basal serum FSH concentration (on day 3 of the menstrual cycle) <12 IU/L; basal serum estradiol concentration <70 pg/mL.

The couples included in our study reported a variety of primary infertility causes, including male factors, tubal factors, endometriosis, and idiopathic causes. Ultrasound examinations revealed uteruses and ovaries of normal size and shape. Semen parameters were evaluated according to the World Health Organization guidelines [23].

All women underwent FSH stimulation and pituitary suppression with an agonist or antagonist of the gonadotropin-releasing hormone (GnRH). The GnRH agonist (leuprolide acetate 0.25 mg/day s.c.) was administered starting from the mid-luteal phase (day 21) of the previous menstrual cycle. After pituitary desensitization, the women were stimulated with recombinant FSH at doses of 225–450 IU/day s.c. The GnRH antagonist (ganirelix acetate 0.25 mg/day s.c.) was administered only after obtaining at least one ≥14 mm follicle in women who had been previously treated from day 3 of the menstrual cycle with recombinant FSH at doses of 225–450 IU/day s.c. FSH was withdrawn after follicular maturation was achieved. Follicular maturation parameters were assessed daily by transvaginal ultrasound (using a Philips HD3 ultrasound system with 6.5-MHz transducer) and serum estradiol assays. As soon as follicular maturation was achieved (with follicles 16–18 mm in diameter and a serum estradiol concentration >1,000 pg/mL), women received 10,000 IU of human chorionic gonadotropin (hCG). Oocytes were retrieved after 34–36 hours by ultrasound-guided transvaginal follicular aspiration using a 17-gauge needle. Oocyte pick-up was carried out under general anesthesia.

As protection in the luteal phase, women were given 200 mg of micronized progesterone vaginally, twice a day, starting from the day before embryo transfer (42–72 hours after oocyte pick-up). Women were considered poor responders when ≤3 oocytes were retrieved or when the ART cycle was cancelled because there was no follicular growth following controlled stimulation. Normal responders had 4–9 oocytes retrieved, while high responders had ≥10 oocytes retrieved or the women had OHSS. Serum samples for the AMH assay were obtained on the first day of stimulation (at baseline) and on the last day of stimulation (the day of hCG administration). Suitable samples were centrifuged for 10 minutes at 2,000 revs and then frozen at −20°C until the AMH assay was performed. AMH was assayed in duplicate using an immunoenzymatic technique (Immunotech, Marseille, France). Assay sensitivity was 0.14 ng/mL; intra- and inter-assay coefficients of variation were ≤12.3% and ≤14.2%, respectively.

Serum estradiol concentrations were assayed in duplicate using a radioimmunological technique with the Coat-A-Count E2 kit (Diagnostic Products Corporation, Los Angeles, CA, USA).

Our data were expressed as means and standard deviations or as frequency rates. Depending on the type of data considered, the statistical analysis was carried out using descriptive statistical methods, Pearson’s correlation test, confidence interval calculation, Student’s t-test for paired data, Spearman’s correlation test and the Mann-Whitney U-test. A P value of ≤0.05 was considered statistically significant.

## Results

Pre-treatment characteristics and demographic data of the women studiedare reported in Table 1^a^. Etiological factors of infertility included male factors (28 cases), tubal factors (9 cases), endometriosis (2 cases), and idiopathic factors (7 cases). Mean AMH values for different etiological factors of infertiulity are reported in Table 1^b^.

**Table 1a:** ^a^ **^,b^.** Pre-treatment features, patient demographic data and mean AMH levels for different etiological factors of infertility.

^a^Pre-treatment features and demographic data	N° patients	Mean	DS
Age (years)	46	35,50	4,09
BMI (kg/m^2^)	46	22,21	2,39
basal FSH (UI/l)	46	8,18	2,64
basal LH (UI/l)	46	4,74	2,02
basal Estradiol (pg/ml)	46	52,40	17,40
basal AMH (ng/ml)	46	4,02	4,36
**^b^Etiological factors of infertilità**	**N° patients**	**Mean AMH levels**	
Male factors	28	4.06	
Tubal factors	9	4.3	
Endometriosis	2	3.4	
Idiopathic factors	7	3.7	

The ART cycle was cancelled in two patients (4.35%) due to absent follicular growth. Embryo transfer was performed in 41 patients (89.13%). Data about ovarian response and reproductive outcome during controlled ovarian stimulation are reported in Table 2.

**Table 2:** Ovarian response and reproductive outcome during ovarian stimulation.

	N° patients	Mean	DS
AMH on day of HCG (ng/ml)	44	1,19	0,82
Days of stimulation	44	9,57	2,76
Total dose of administered FSH (IU)	44	2298,30	931,11
Estradiol on day of HCG (pg/ml)	44	1798,30	1068,52
Follicles on day of HCG	44	10,91	5,93
Collected oocytes	41	5,07	2,742
Fertilized oocytes	41	3,41	2,27
Obtained embryos	41	2,29	1,65
Transferred embryos	41	2,10	1,41

No significant correlation was found between basal AMH and a woman’s age, basal LH and basal estradiol levels (p: n.s.); while the correlation between basal AMH and basal FSH levels is statistically significant (p: 0.007). In fact, FSH levels (8.78±4.04) increased significantly when AMH levels decreased (ρ<0; P<0.01) (Table 3^a^).

**Table 3:** Correlations between AMH serum level and pre/post treatment features.

	N° patients	ρ	p
^a^Age	46	−0,224	0,134
^a^Basal LH	46	−0,038	0,808
^a^Basal estradiol	46	−0,093	0,623
^a^ **Basal FSH**	**46**	**−0,400**	**0,007**
^b^Total dose of administered FSH (IU)	44	−0,285	0,064
^c^ **Follicles on day of HCG**	**44**	**0,662**	**<0,001**
^c^ **Estradiol on day of HCG**	**44**	**0,548**	**<0,001**
^c^ **Collected oocytes**	**41**	**0,643**	**<0,001**
^d^ **Fertilized oocytes**	**41**	**0,400**	**0,009**
^d^Obtained embryos	41	0,294	0,062
^d^Transferred embryos	41	0,289	0,067

Notes:

a) Correlation between AMH and pre-treatment features of patients;

b) Correlation between AMH and total dose of administered FSH;

c) Correlation between AMH and ovarian response;

d) Correlation between AMH and reproductive outcome.

The total dose of administered recombinant FSH to induce ovulation (2,298±931.11) was not correlated to AMH (p: n.s.) (Table 3^b^). Ovarian response, distinguished by the number of follicles on the hCG day (10.91±5.93), serum estradiol levels on the hCG day (1,798.30±1,068.52), and the number of retrieved oocytes (5.07±2.74) was significantly correlated to AMH (ρ >0; P<0.01) (Table 3^c^). The number of fertilized oocytes (3.41±2.27) was also significantly correlated to AMH (ρ>0; P<0.01). No significant correlation was found between obtained embryos (2.9±1.65) or transferred embryos (2.10±1.41), and AMH (P = n.s.) (Table 3^d^).

**Figure 1:**
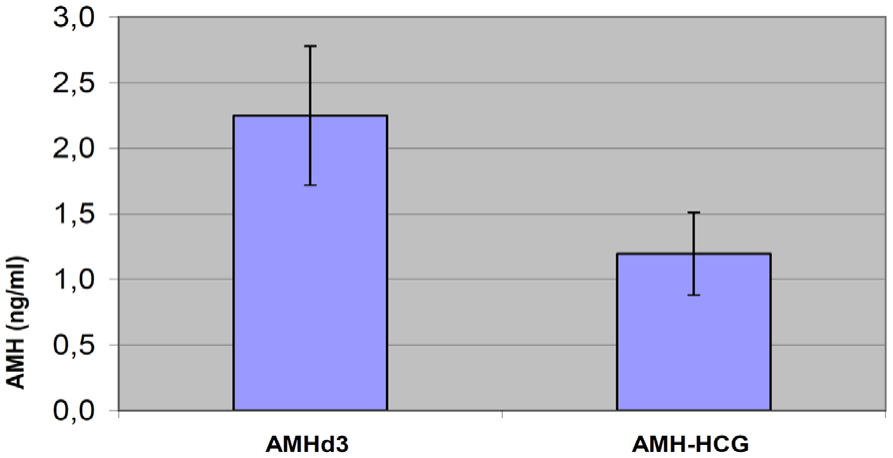
Values of serum AMH: baseline and on day of hCG administration level. Notes: AMHd3 = AMH on the 3^th^ day of the menstrual cycle (baseline). AMH-HCG = AMH on the day of hCG administration. T test for paired data. AMHd3 vs AMH-HCG: t = 5,484; p<0,001.

Basal serum AMH levels (on day 3 of the menstrual cycle) were significantly higher than AMH levels measured on the hCG day (p<0.01) (Fig. 1).

**Figure 2:**
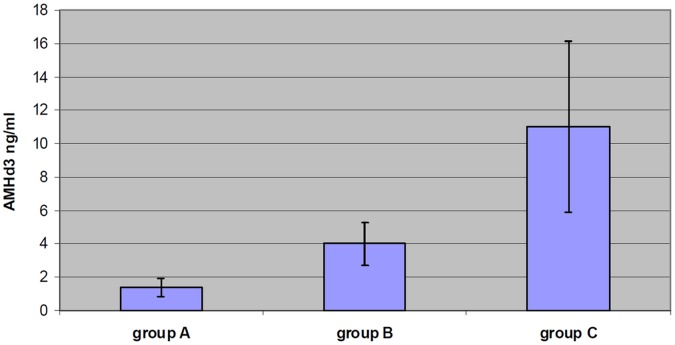
values of AMHd3 and their 95% confidence intervals according to response group. Notes: group A  =  poor responders; group B  =  normal responders; group C  =  high responders; AMH group B vs AMH group A: P<0,001; AMH group B vs AMH group C: P<0,001.

The AMH values in the different groups of responders to ovarian stimulation are reported in Table 4. There was a significant correlation between AMH in normal responders and AMH in both high and poor responders (Fig. 2). The correlation of AMH between poor and high responders was also significant as reported in Table 5.

**Table 4:** AMH levels in different response groups to ovarian stimulation.

	Poor responders(≤3 obtained oocytes/cancellationof the cycle)	Normal responders(4–9 obtained oocytes)	High responders(≥10 obtained oocytes/OHSS)
**N° patients**	15	25	6
**Mean**	1,4	4	11
**DS**	1,1	3,2	6,4
**Minimum**	0	0,91	2,58
**Maximum**	3,45	14,37	21
**Median**	1,25	3,09	11,01

Notes: OHSS = ovarian hyperstimulation syndrome.

**Table 5:** Mann-Whitney test for comparison between poor responders and high responders.

	Z	P
AMH group A vs. AMH group C	−3,23	<0,001

Notes: group A  =  poor responders; group C  =  high responders.

## Discussion

Our study confirmed the usefulness of AMH as a biomarker of ovarian function. We showed an inverse correlation between serum AMH and serum FSH levels measured on the third day of the menstrual cycle. We also noted that high levels of AMH were positively correlated with the number of retrieved and fertilized oocytes, which are all indicators of ovarian function.

Many other studies suggest AMH as a novel measure of ovarian reserve. AMH levels decrease throughout a woman’s reproductive life [15], [24]. Serum levels on day 3 of the menstrual cycle show a progressive decrease with age, which correlates with AFC [15]. Undetectable AMH levels after spontaneous menopause have been reported [14], [18], [24]. Ovariectomy in regularly cycling women is associated with the disappearance of AMH in 3–5 days, demonstrating that circulating AMH is exclusively of ovarian origin [14], [24]–[26]. AMH is an endocrine marker that reflects the transition of resting primordial follicles to growing follicles. AMH declines gradually in the 5 years prior to the final menstrual period, perhaps representing a critical biological juncture during the transition to menopause [2].

Once again, AMH is the best indicator of ovarian health, and therefore it is a crucial parameter for IVF success.

In Western societies, the introduction in the 1960s of reliable methods of contraception has led to the birth of fewer children per family. Driven by increasing levels of female education, a growing participation in the labour force and career demands, postponement of childbearing has been a secondary consequence of the so-called sexual revolution [27]. These societal changes in family planning have caused a significant increase in the incidence of unwanted infertility due to female reproductive ageing [27]–[30]. The reduction in female fertility has also be shown in contemporary population studies. The chance of not conceiving a first child within one year increases from under 5% in women in their early 20 s to approximately 30% or more in women aged 35 and older^29^. So, although the majority of older women will become pregnant within a one-year period, the chance of becoming subfertile increases about sixfold in comparison with very young women [27].

Precisely for these reasons, the use of IVF has grown exponentially in Western countries. Nonetheless, the probability of live births obtained through IVF treatment clearly decreases after age 35 [31].

The same is true for the implantation rate per embryo [32]. In fact, female age has consistently been shown to be an important predictor of successful IVF treatments [27].

Generalizing in medicine is incorrect since we all know that biological parameters vary widely from individual to individual. For this reason, we tried to find a biological parameter that would allow gynecologists to better identify patients’ ovarian quality in order to choose the most appropriate drug treatments. This biomarker appears to be AMH.

The parameters we studied to investigate the correlation between AMH and ovarian response were follicle count and estradiol concentration per day of hCG, and the number of retrieved oocytes. We found a significant correlation between AMH and these parameters.

The AMH value as a predictor of ovarian response in controlled ovarian stimulation cycles was confirmed in several studies [1], [15]–[21], which demonstrated a significant correlation both with the number of follicles on the hCG day [1], [33] and with the number of retrieved oocytes [16], [34], [35].

Granulosa cells of primary follicles show homogeneous AMH expression; in larger follicles, AMH is mainly produced in cells near the oocyte and in a few cells surrounding the antrum. AMH continues to be expressed in growing follicles in the ovary until they have reached adequate size and state of differentiation to be selected for dominance by the action of pituitary FSH. In the mouse this occurs at the early antral stage in small growing follicles [9], while in women in antral follicles of size 4–6 mm [11], [12]. AMH is not expressed in atretic follicles and theca cells [11], [12], [24], [36]–[39].

AMH levels therefore represent a useful indicator of the number of follicles in the early stage, which are transformed into larger follicles during controlled ovarian stimulation as a result of exogenous FSH administration [17].

In our study we found a significant correlation between AMH values and responder groups (poor responders, normal responders and high responders). AMH values were higher in high responders and lower in poor responders. Knowing the baseline values of AMH allowed us to choose the amount of FSH to be administered to each patient, thus saving on government spending and getting the best response from women. For example, a young woman with diminished ovarian reserve (based on AMH values) may be given preference on the waiting lists of public treatment centres (ranging from 6 to 12 months) to start of the first IVF cycle and the IVF specialist may choose for this woman a stronger-than-usual stimulation treatment.

OHSS seems to be associated with significantly higher basal AMH levels [1], [40], [41]. This suggests its clinical usefulness in preventing an excessive response.

In our study, AMH did not appear to influence reproductive outcome. However, this finding does not seem important because our protocols were not affected by basal AMH values, as we chose to assess them only after embryo transfer. This must make us reflect on whether we should use customized protocols to improve pregnancy rates.

Another finding of our study, which is somewhat at variance with the literature but is nonetheless extremely important, is the inverse relationship between basal AMH and the hCG day. As the AMH measurement was done before administering hCG, the unexpected results cannot be attributed to massive luteinization of the follicles, which might explain the fact that serum AMH levels do not significantly change throughout the menstrual cycle [42].

By contrast, in our study, baseline AMH values were significantly higher than those of AMH on the hCG day. The initial purpose of our measurement was to confirm this fact. What we found instead were unexpected results, which we will continue to assess in other patients and hopefully will be able to publish soon. Such results are not easily explained.

A few authors believe that AMH levels gradually decrease during ovarian stimulation due to a strong decrease in the number of small antral follicles, associated with the progressive increase in the number of larger follicles [43]. Several studies conducted in rats [39], [44] and on the ovarian tissue of adult women [11] showed decreased expression levels of AMH in larger antral follicles compared to smaller ones. Albeit valid, this theory cannot explain the significant decrease that we found in AMH levels. Such a significant reduction cannot be related to an increase in preovulatory follicles, especially because prenatal follicles continue to be expressed, sometimes in large numbers.

A recent study showed that the highest level of AMH expression was found in the granulosa cells of secondary, preantral and small antral follicles <4 mm in diameter. In larger (4–8 mm) antral follicles, AMH expression gradually disappeared [11]. Moreover, early follicle growth in humans appears to be independent of stimulation by gonadotropins [45].

We are currently conducting a prospective trial on the advisability to reconsider AMH concentrations after about 60 days to determine if the decrease in hormone levels reduction is transient or not. In the case that it is intransient, with each IVF cycle we should see a dramatic reduction of ovarian reserve and we should reconsider the maximum number of stimulations to prevent premature menopause.

The reduction in AMH levels observed during FSH administration may be due to a negative role of FSH on AMH secretion. Indeed, it is well established that FSH is a positive regulator of testicular AMH gene expression [46], whereas other Authors [39] previously reported that FSH may down-regulate AMH and AMH type-II receptor (AMHRII) expression in adult rat ovaries. Alternatively, the reduction in AMH levels could be due to the supraphysiological increase in estradiol levels observed when exogenous FSH is administered. Estradiol has been implicated in the down-regulation of AMH and AMHRII mRNA in the ovary [24], [39]. Therefore, this finding is not clear and needs to be confirmed by further studies.

In conclusion, our study suggests that the clinical usefulness of AMH in ART cycles consists in the possibility of customizing treatment protocols. This would make it possible for patients to entertain more realistic expectations and minimize both the psychological stress related to poor response or cycle cancellation and OHSS-related morbidity.
